# Adoptive T Cell Therapy Targeting CD1 and MR1

**DOI:** 10.3389/fimmu.2015.00247

**Published:** 2015-05-20

**Authors:** Tingxi Guo, Kenji Chamoto, Naoto Hirano

**Affiliations:** ^1^Department of Immunology, University of Toronto, Toronto, ON, Canada; ^2^Princess Margaret Cancer Centre, University Health Network, Toronto, ON, Canada

**Keywords:** immunotherapy, CD1, MR1, NKT, MAIT, T cell, adoptive cell transfer

## Abstract

Adoptive T cell immunotherapy has demonstrated clinically relevant efficacy in treating malignant and infectious diseases. However, much of these therapies have been focused on enhancing, or generating *de novo*, effector functions of conventional T cells recognizing HLA molecules. Given the heterogeneity of HLA alleles, mismatched patients are ineligible for current HLA-restricted adoptive T cell therapies. CD1 and MR1 are class I-like monomorphic molecules and their restricted T cells possess unique T cell receptor specificity against entirely different classes of antigens. CD1 and MR1 molecules present lipid and vitamin B metabolite antigens, respectively, and offer a new front of targets for T cell therapies. This review will cover the recent progress in the basic research of CD1, MR1, and their restricted T cells that possess translational potential.

## Introduction

Given their central role in numerous diseases, T cells have become the focus and mediators of many immunotherapies. T cell immunotherapy has had impressive success in treating malignant and infectious diseases. Currently, there are several methods of using T cells as therapy. T cells are cultured and/or engineered *ex vivo* and adoptively transferred into the patient, or T cells are directly targeted *in vivo* by vaccination or biological compounds. Regardless of the approach taken, these immunotherapies generate a *de novo* T cell-mediated immune response and/or enhance preexisting functions, which are often suppressed in patients. Adoptive T cell transfer therapy offers unique advantages and has been considerably tested in numerous trials. The modern approach to this method allows the personalization of T cells through the desired *ex vivo* activation, expansion, and genetic modification, followed by infusion back into the patient (1). As part of this method, we are able to produce a large number of long-lived memory T cells with defined functions, which last up to years in the patients after infusion depending on the expansion protocol (2). The T cells can also be genetically engineered to express recombinant T cell receptors (TCRs) or chimeric antigen receptors (CARs) to specifically target tumor or pathogen-associated antigens. Whereas CARs are only able to target surface molecules, TCRs recognizing peptide antigens presented on HLA are able to target the large repertoire of intracellular antigens.

**Figure 1:**
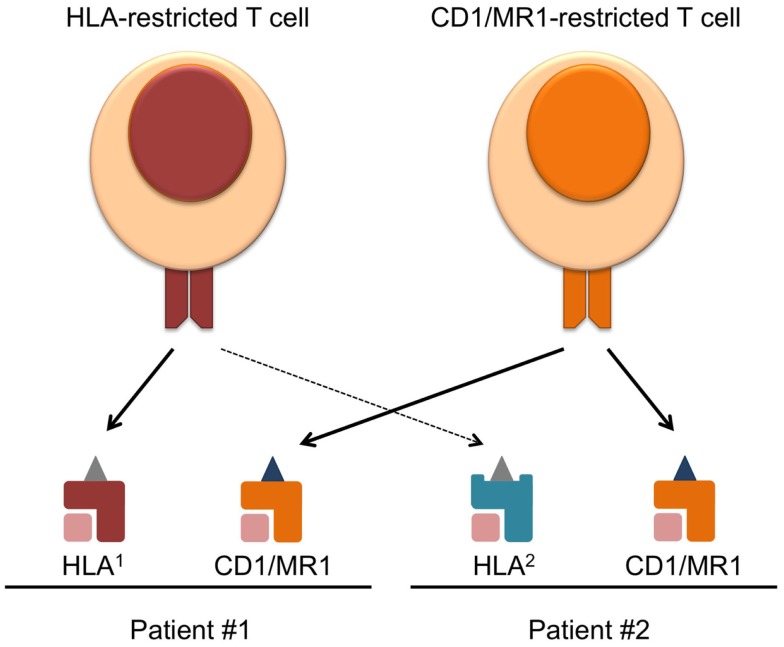
**Overcoming HLA-restriction of adoptive T cell therapy by targeting monomorphic CD1 and MR1**. Current T cell therapies targeting HLA–peptide complexes only benefit patients expressing the compatible HLA allele, which limits its applicability. CD1 and MR1 are monomorphic antigen-presenting molecules, and T cell recognizing CD1/MR1 can target the same antigen complex in patients expressing different HLA. Requirements for the success of such therapy and challenges faced in the field are discussed in the text.

In the past and current TCR-directed adoptive T cell transfer therapies, most trials have been focused on conventional T cells restricted to one HLA allele. The human HLA gene locus is vastly varied between individuals (3), and although conventional T cell therapies have aimed to target common alleles such as HLA-A2, a significant portion of HLA-mismatched patients cannot benefit from this type of treatment. Therefore, the heterogeneity of HLA alleles represents a major barrier to the applicability of current TCR-directed adoptive T cell therapies. With the recent advancements in the field of CD1, MR1, and their, respectively, restricted T cells, these molecules are becoming attractive targets of immunotherapy. These molecules offer the advantage of being monomorphic antigen-presenting molecules that are conserved across humans, as well as the ability to present completely different classes of antigens other than peptides (4). Therefore, targeting CD1 and MR1 will broaden the applicability of adoptive T cell therapy (Figure 1).

The MHC class I homolog CD1 family of molecules contains four antigen-presenting members in humans, CD1a–d, and only one in mice, CD1d (5). Many of the CD1 studies have been focused on invariant natural killer T (iNKT) cells (type I NKT) found in both humans and mice. These cells are defined by their invariant TCRα and semi-variant TCRβ gene usage, and the recognition of the canonical ligand, α-galactosylceramide (α-GalCer) (6). The nature of type II NKT cells, which comprise the remainder of CD1d-restricted T cells that do not recognize α-GalCer, and CD1a–c-restricted T cells have become better understood in recent years. MR1 is also an MHC class I homolog presenting vitamin B metabolites. MR1–antigens complexes are recognized by mucosal-associated invariant T (MAIT) cells, which are another group of evolutionarily conserved αβ T cells found in high numbers in humans (7). Like iNKT cells, they express an invariant TCRα chain that is paired with an oligoclonal TCRβ chain repertoire (8). These molecules and cognate T cells will be discussed further in details below.

To date, the only clinical trials involving CD1 and MR1 have been utilizing iNKT cells as a cellular adjuvant by activating them via α-GalCer. Many mouse studies implicated roles for iNKT cells in tumor regression (9) and antimicrobial immunity (10). Unfortunately, many of these findings have not been translated well to humans. In the published clinical trials, when cancer or chronically infected patients were treated with iNKT cells activated by α-GalCer, alone or pulsed on antigen-presenting cells (APCs), only limited efficacy was observed (11–19). Based on the experiences that led to effective adoptive T cell therapy targeting HLA, T cell therapies targeting CD1 and MR1 can be improved. In this review, we will address how CD1 and MR1 can be targeted more effectively in diseases by examining the three constituents of successful adoptive T cell transfer therapy, which are the knowledge of (1) disease-associated target antigen complexes, (2) TCRs that recognize these complexes specifically without eliciting harmful autoimmunity, and (3) the optimal function of the responding T cells.

## The Targets

To target CD1 or MR1 in diseases, their expression on the pathological tissue of interest is necessary. However, the presence of antigen-presenting molecule alone is not enough. An effective T cell therapy should ideally target diseased tissue specifically, with minimal autoimmune response against healthy tissues. Therefore, understanding the nature of antigens presented during pathological and steady state is required to safely and efficiently target CD1 and MR1 in diseases (Table 1).

**Table 1:** **Characteristics of CD1 and MR1 antigen-presenting molecules and their, respectively, restricted T cells in humans**.

Antigen-presenting molecule	Pattern of surface expression	Nature of antigens presented	Cognate TCRs	Frequency of cognate T cells
CD1a	Restricted [thymocytes, professional APCs, Langerhans cells, Ref. (20)]	Mycobacterial lipopeptide, and self apolar lipids (21, 22)	Diverse TCRs	~Up to 20% of CD4^+^ and CD4^−^CD8^−^ T cells (23, 24)
CD1b	Restricted [thymocytes, professional APCs, Ref. (25)]	Mycobacterial lipids (26, 27)	GEM [TRAV1-2–TRAJ9, Ref. (28)], and diverse TCRs	~Up to 1.5% of CD4^+^ and CD4^−^CD8^−^ T cells (23)
CD1c	Restricted [thymocytes, professional APCs, Ref. (25)]	Mycobacterial lipids and self lysophospholipid (29, 30)	Diverse TCRs	~Up to 7% of CD4^+^ and CD4^−^CD8^−^ T cells (23)
CD1d	Widely expressed [e.g., hematopoietic, gastrointestinal, and reproductive tissues, Ref. (31, 32)]	Bacterial and self glycolipids, plasmalogens, phospholipids (33–44)	iNKT (mostly TRAV10– TRAJ18 paired with TRBV25), and diverse TCRs (38)	~Up to 3% of CD4^+^ and CD4^−^CD8^−^ T cells (23)
MR1	Unknown [widely expressed at the mRNA level, Ref. (45)]	Small molecule metabolites (46, 47)	TRAV1-2 paired with TRBV20 or TRBV6 TCRs (48)	~1–10% of total T cells (48, 49)

*Antigens listed only include those that have been identified and validated. Frequency is among peripheral blood T cells in healthy humans*.

### Pattern of expression of CD1 and MR1

The CD1 family of lipid-presenting molecules can be separated based on patterns of expression into two groups. Group 1 includes CD1a, CD1b, and CD1c, and is mainly found on professional APCs and developing thymocytes, with CD1a more strictly restricted to Langerhans cells (20, 25, 50). Group 2 only includes CD1d and is expressed widely on many tissues (31, 32). Similar to CD1d, MR1 is expressed at the transcript level in many tissue types (45), but the detection of surface MR1 expression has been challenging. A newly developed mAb against murine MR1 was able to stabilize and enhance its transient surface expression (51). However, the MR1 surface expression pattern on human healthy and pathological tissues is largely unknown.

All CD1 molecules can be found on various leukemia and lymphomas, although the exact expression pattern varies between patients (29, 52, 53). In addition, CD1d can be found on subsets of medulloblastoma, multiple myeloma, and renal cell carcinoma patients (54–56). Other cells in the tumor microenvironment that suppress anti-tumor immunity and/or promote tumor growth also express CD1 molecules. For example, tumor-associated monocytes and macrophages, which are associated with poor prognosis in neuroblastoma patients, were found to express CD1d and could be targeted by iNKT cells in a mouse model (57). Targeting CD1d on both the tumor cells and the supporting stromal cells could be an effective approach.

In infections, CD1d expression can be downregulated by viral immune evasion mechanisms of human immunodeficiency virus and herpes simplex virus (58, 59). Similar to MHC class I, surface CD1d expression on epithelial and immune cells is upregulated in inflammatory conditions and can be induced by interferons (60). Yakimchuk et al. recently demonstrated that CD1b and CD1c molecules are upregulated on Langerhans cells of Lyme disease patients compared to healthy samples. *In vitro*, all group 1 CD1 molecules on monocytes could be upregulated by stimulation with extracted *Borrelia burgdorferi* lipids via TLR-2 as well as IL-1β (61). The expression of CD1 and MR1 molecules need to be better characterized for different diseases, since understanding the surface expression pattern on infected or transformed cells *in vivo* is essential for CD1- or MR1-restricted immunotherapy.

### Antigens presented by CD1 and MR1

Several studies have shed light on the disease associated and natural antigens presented by CD1 molecules. The ability of group 1 CD1 molecules to present foreign mycobacterial antigens, such as dideoxymycobactin, glucose monomycolate, and mycolic acids, has been well established (21, 26, 27, 30). These antigens are uniquely derived from the bacteria and are not found in the absence of infection. Lepore et al. discovered a novel tumor-associated self-lipid antigen presented by CD1c. Methyl-lysophosphatidic acid was found 100-fold higher in acute B lymphoblastic leukemia and acute myeloid leukemia cells compared to normal B cells or monocytes, and stimulated T cells in a CD1c-dependent manner (29). Although this lipid was also found at elevated levels in dendritic cells, it nevertheless demonstrated the existence of tumor-associated lipid antigens. In addition, some studies have characterized changes in the lipidome of transformed cells (62, 63). Therefore, it is likely that more tumor-associated lipid antigens exist and possibly shared across different patients and cancers. The nature of antigenic self-lipids presented by CD1a were also recently elucidated by de Jong et al. Using a CD1a-restricted T cell line, the group showed that apolar lipids lacking hydrophilic functional groups such as squalene and triacylglyceride were antigenic when presented by CD1a (22).

CD1d is able to present several microbial derived α-linked glycolipids, which are potent activators of iNKT cells (33, 34). For virus-derived lipid antigens, none have been identified to date. However, Zeissig et al. demonstrated that hepatitis B virus-infected human hepatocytes stimulated iNKT cell lines significantly more than non-infected, and this was attributed to the enrichment of virus-induced endogenous antigenic lysophospholipids (35, 36). Several new stimulatory lipid ligands recognized by type II NKT cells have also been discovered. Tatituri et al. demonstrated that bacterial and mammalian phosphatidylglycerol and diphosphatidylglycerol were able to stimulate murine type II NKT hybridomas, but not iNKT cells (37). Nair et al. identified β-glucosylceramide and glucosylsphingosine lyso-glucocerebroside as antigens for human and mouse type II NKT cells. The circulating levels of these two lipids are elevated in patients with Gaucher’s disease. The group stained PBMC with CD1d tetramer loaded with either of the two lipids, and found that the tetramer positive cells did not express the invariant NKT TCR, indicating that they were not type I iNKT cells. Their frequencies were, in fact, much higher than α-GalCer tetramer positive cells. Monocyte-derived DCs pulsed with each antigen were able to expand the respective tetramer positive population (38), validating their stimulatory capacity for type II NKT cells.

In terms of steady state self-lipid antigens, iNKT cells recognize phospholipids, plasmalogens, and glycolipids (39–41). Until recently, it was thought that endogenous mammalian and foreign bacterial glycolipids differed in the β or α linkage between the sugar moiety and the lipid. This was due to the fact that mammals lack the enzyme required to form α-linked glycolipids, which allowed iNKT cells strongly recognizing α-linked glycolipids to easily distinguish between self and foreign antigens. However, two independent groups have reported that a previously identified endogenous β-linked glycolipid, β-glucopyranosyl ceramide (42), was contaminated with the rare, but strongly stimulatory α-linked version (43, 44). Kain et al. demonstrated using α-linked glycolipid specific antibody that mammalian cells likely produced this class of lipids (44). These findings strongly influence the choice of antigen for CD1d targeted therapy, since the previously assumed structural exclusivity of foreign and self-glycolipids may not be so strict.

Mucosal-associated invariant T cells are activated by bacteria but the nature of microbial antigens presented by MR1 remained elusive until recently. Kjer-Nielsen et al. demonstrated vitamin B metabolites as putative ligands presented by MR1, and solved the x-ray crystal structure of a folic acid derivative (6-FP) complexed with MR1. This study also showed that, although 6-FP was not immunogenic in stimulating MAIT TCR transfectants *in vitro*, a riboflavin-derivative isolated from the supernatant of *Salmonella* culture was able to upregulate MR1 expression on an MR1-expressing target cell line and activate primary human MAIT cells (46). The same group also identified byproducts of an intermediate of the riboflavin synthesis pathway as MAIT antigens (47). Given the strong influence of gut microbiota on MAIT cells (64), addressing whether these identified antigens are presented only during infection or also at steady state dictates their therapeutic potential.

## The Receptors

The responsible molecule that targets CD1 or MR1 is the TCR. Clinical trials have confirmed that T cells engineered to express a recombinant TCR can effectively target cells expressing cognate antigens in humans. Therefore, understanding the repertoire of CD1 and MR1-restricted TCRs (Table 1) and their molecular mechanism of antigen recognition are important for developing therapy directed at the molecule of interest.

### CD1- and MR1-restricted invariant T cell receptors

Invariant NKT TCRs are the CD1-restricted TCRs that have been most extensively characterized. In humans, iNKT TCRs are largely composed of the TRAV10–TRAJ18 invariant TCRα chain paired with TRBV25 TCRβ chains with a hypervariable CDR3β region. In mice, they are mainly TRAV11–TRAJ18 TCRα paired with TRBV13, 29, or 1 TCRβ chains (4). Non-TRAV10 and non-TRAV11 iNKT cells have also been identified in humans and mice, respectively, but are generally rare (65, 66). As mentioned above, successful T cell therapy relies on maximizing on-target effect, while minimizing off-target autoreactivity. Having disease-specific antigen is only half of the requirement, since antigen-specific receptors are also required to distinguish them from normal tissue antigens. Several studies demonstrated that iNKT TCRs act as pattern-recognition receptors, unable to distinguish the lipid antigen presented by CD1d (67–69). This is attributed to its conserved docking mode on CD1d, where only the germline-encoded regions of the TCR are involved in recognizing the lipid antigen, while the single variable region, CDR3β, interacts with the antigen-presenting molecule (67, 68, 70). Therefore, the diversity in the TCR is supposed to only impact the overall affinity to the lipid-CD1d complex, but not antigen selectivity. This would prevent the isolation of tumor or pathogen-specific iNKT TCR. However, our group has recently characterized a large panel of natural human iNKT TCRs and demonstrated selective antigen recognition of different lipid–CD1d complexes. Furthermore, it appears that most of the peripheral human iNKT cells express antigen-selective TCRs (in revision, Chamoto et al.). Given the many crystal structures of iNKT TCR–CD1d complexes showing the same docking mode, these newly identified human iNKT TCRs unlikely possess an alternate docking mode. It is possible that the hypervariable CDR3β adjust the conformation of the antigen-recognizing germline portion of the TCR (71), in a sequence-dependent manner, thus allowing for distinction between antigens. Intriguingly, mouse and human iNKT TCRs possess cross-species reactivity for human and mouse CD1d, respectively (72). It remains to be seen whether mouse iNKT TCRs are able to distinguish lipid antigens presented by human CD1d. HLA-restricted mouse TCRs have been already tested in the clinic without causing any toxic xenoreaction in cancer patients (73).

Two other subsets of T cells expressing an invariant TCRα chain have been characterized. Recently identified germline-encoded, mycolyl lipid-reactive (GEM) T cells are a subset of CD1b-restricted T cells expressing a TRAV1-2–TRAJ9 TCRα chain. These TCRs possess a fixed CDR3α length and minor amino acid variations across different tuberculosis patients. Clonotypic GEM TCRs recognized either mycobacterial antigen glucose monomycolate or mycolic acid presented by CD1b. Structural analysis demonstrated that the footprint of GEM TCRs on CD1b–antigens resembled conventional TCRs, which explains their ability to distinguish the two antigens. Furthermore, GEM TCRs recognized foreign antigens with high affinity and did not display baseline autoreactivity (28). Thus, they represent a viable option in targeting these mycobacteria-derived antigens.

Mucosal-associated invariant T cells represent the third group of T cells with a biased TCR repertoire. Recently developed MR1 tetramer loaded with the stimulatory riboflavin-derivative demonstrated that MAIT TCRs also utilize the TRAV1-2 gene, which is mostly rearranged to TRAJ33, and pairing mostly with TRBV20 or TRBV6-4 (48). Functional and structural studies on MAIT TCRs suggested that they possess antigen selectivity, where stimulation by MR1^+^ target cells infected by different genus of microbes specifically enriched different clonal populations of MAIT cells *ex vivo* (74). This is consistent with the crystal structures of MAIT TCR–MR1–antigen complexes, where the TCR takes a more perpendicular docking mode similar to GEM and conventional TCRs (75, 76). In these studies, no interactions were identified between the hypervariable CDR3β of the MAIT TCR and the two vitamin B metabolite antigens previously identified. Interestingly, however, this docking mode permitted the hypervariable region of the MAIT TCR to interact with novel antigens (47) and a derivative of 6-FP presented by MR1 (77). In the two subsequent studies, the crystal structures of several MAIT TCRs–MR1–antigen complexes were solved, showing that the CDR3β loop directly interacted with the antigen. Collectively, these evidences support an antigen-selective mode of recognition by MAIT TCRs.

### CD1-restricted diverse T cell receptors

Non-invariant TCRs recognizing CD1 represent the majority of the total CD1-restricted TCR repertoire in humans (23, 38). These diverse TCRs do not appear to possess the conserved parallel docking mode seen with iNKT TCRs. The crystal structures of murine type II NKT TCRs recognizing CD1d-self-antigens demonstrated an orthogonal docking mode similar to the one classically seen with MHC-restricted TCR (78, 79). The CDR3β made direct interactions with the antigens, indicating that type II NKT TCRs, if all similarly possess this docking mode, would potentially be able to discriminate antigens depending on the hypervariable CDR3 sequences. Roy et al. performed alanine scanning on the CD1c molecule presenting mycobacterial phosphomycoketide (PM) and measured the dissociation constant for the mutants against a panel of CD1c–PM reactive clones (80). The group observed that different point mutations affected the strength of interaction differently for different TCRs. This would not be expected if all the TCRs recognized the CD1c complex in a conserved manner. Whether this variable docking mode holds true for CD1c presenting other foreign and self-antigens remains to be tested, and examining the antigen selectivity of this unpredictable docking mode requires experiments involving more antigens. The crystal structure of TCR–CD1a–self ligand was recently solved with a clonotypic CD1a-restricted TCR. In this study, although the TCR docked orthogonally onto CD1a, the recognition of the antigen complex relied on contacts with CD1a only. This allowed the TCR to recognize various “permissive” self-ligands that did not disrupt the TCR–CD1a interaction (81). It will be of interest to see if this is also the case with other CD1a-restricted TCRs. How diverse CD1b-restricted TCRs recognize the cognate CD1–antigen complex and their ligand selectivity remains uncharacterized. CD1c- and CD1d-restricted γδ TCRs have also been identified (82–84), and offer a separate repertoire from which to isolate disease-specific receptors. Structural analysis demonstrated that CD1d-restricted γδ TCRs recognized the antigen complex also similarly to conventional MHC-restricted TCRs (85). The germline-encoded CDR1 and CDR2 recognized the monomorphic CD1d, while the hypervariable CDR3δ was positioned on top of the ligand. This is highly suggestive of an antigen-selective mode of recognition. The wealth of potentially antigen-selective αβ and γδ TCRs recognizing CD1- or MR1-antigen complexes hold great therapeutic potential for cancer and infection-specific T cell therapy restricted by CD1 or MR1.

## The Cells

### Functional phenotype of CD1- and MR1-restricted T cells

In the cases of cancer and infection, the ultimate goal of immunotherapy is to induce cell death in the malignant or infected cells to control the disease. As mentioned above, iNKT cells have been targeted with the aim to jump-start the ensuing immune response that ultimately leads to a cytotoxic cellular response. However, in mice, multiple functional subsets of iNKT cells paralleling MHC class II-restricted T cells have been discovered. Lee et al. identified NKT1, NKT2, and NKT17 subsets that preferentially secrete IFN-γ, IL-4, and IL-17, respectively, based on the lineage transcription factor expressed (86). It is unknown whether the NKT1, NKT2, and NKT17 functional subsets exist in humans, and if so, the variation in the frequency of the different functional lineages between individuals needs to be addressed. A small fraction of suppressive IL-10 secreting iNKT cells was reported in humans, and their frequency in peripheral blood ranged over one order of magnitude between individuals (87). Activation of one or a few particular functional subsets of iNKT cells by α-GalCer in some of the clinical trials might explain the lack of efficacy, especially if the activated subset antagonizes a favorable response. As for the other CD1-restricted T cells and MR1-restricted MAIT cells, their functions *in vivo* are largely unknown but likely resemble Th1 and/or Th17 phenotypes. *Ex vivo* or *in vitro* stimulation studies demonstrated that non-iNKT CD1-restricted T cells are capable of producing IFN-γ and MAIT cells producing both IFN-γ and IL-17, both with some capacity for cytotoxicity and IL-2 production (22, 23, 28, 61, 74, 88–90). CD1a-restricted T cells are also able to produce IL-22, consistent with their role in dermal immunity (24). Nevertheless, the fidelity of much of these functions *in vivo* remains to be examined before CD1-restricted T cells and MAIT cells can be used to combat the appropriate disease. Although a Th1 functional profile is generally preferred for optimal anti-tumor and viral immunity, the multifaceted functionality of CD1- and MR1-restricted T cells can potentially expand their applicability to other diseases.

### Large scale production of effector cells

Once the function of these cells has been established, other aspects of making adoptive therapy successful need to be considered. Obtaining a large number of effector cells is important for efficacy. For example, typically 10–100 billion HLA-restricted T cells are infused to a single cancer patient, although not all are antigen-specific, it still represents a sizable number. Depending on the type of function and the aim of the therapy, fewer cells could be required. Nevertheless, given the limited number of iNKT cells in the periphery, it will be necessary to expand this population for therapy. α-GalCer-based stimulation has been traditionally used and serves as an effective method to expand iNKT cells, either pulsed on APCs or in a cell-free system. However, this method expands all iNKT cells independent of antigen selectivity, avidity, and functional profile. There is also evidence indicating that α-GalCer-expanded iNKT cells possess an anergic phenotype (91). Although other CD1-restricted T cells and MAIT cells are more numerous in the peripheral blood (23, 24, 48, 49), they will likely also require *ex vivo* expansion for therapeutic use (Table 1). Cell-based artificial antigen-presenting cells (aAPCs) have been highly effective in expanding conventional MHC-restricted T cells in preclinical and clinical settings (2, 92–94). Similar aAPCs expressing different CD1 molecules have been developed and could stimulate, respectively, restricted T cells (24). It is possible to improve the capacity of these aAPCs to expand and stimulate CD1 or MR1-restricted T cells by co-expressing the necessary costimulatory molecules along with CD1 or MR1, and culturing in the appropriate cytokine milieu (92).

### Memory and longevity

The longevity of infused T cells *in vivo* is also an important factor in clinical success. It has been demonstrated that central memory T cells are more effective than terminally differentiated T cells in adoptive T cell models of cancer and chronic viral infection, owing to their prolonged survival and effector output (95). iNKT cells are well-known for their pre-primed effector memory phenotype, immediate after maturation and in the absence of antigen exposure, which allows their rapid response. Majority of iNKT cells do not express L-selectin (6), a key marker of naïve and central memory phenotype. Although the *in vivo* turnover rate of peripheral and adoptively transferred iNKT cells is unknown, their effector memory phenotype does not suggest prolonged persistence to the extent of central memory conventional T cells. By contrast, a significant portion of type II NKT cells were recently described to possess a naïve-like phenotype, expressing L-selectin and CD45RA (38). This strongly suggests that they could potentially differentiate to long-lived central memory T cells. In fact, CD1-restricted T cells in general, excluding iNKT cells, seem to possess a naïve T cell phenotype (23). Likewise, MAIT cells in PBMC express central memory markers and start to demonstrate a memory phenotype as early as the age of 3 months in humans (89). Whether the expression pattern of memory phenotype markers on these unconventional T cells confer the same cellular longevity as conventional MHC-restricted T cells need to be evaluated. Furthermore, the memory phenotype of CD1- and MR1-restricted T cells can be altered during *ex vivo* expansion, as seen with conventional T cells, where they lose their survival capacity after repeated stimulation and subsequent expansion (96). The aAPC system developed can be a useful tool to expand these cells, while maintaining the desired memory phenotype (2, 97).

## Mix and Match – Building the Best Therapy

In the age of genetic engineering reaching ever higher levels of feasibility and safety, cellular immunotherapies are no longer limited by the inherent constraints of the naturally existing immune system. The aforementioned problems of iNKT cells in therapy (e.g., apparent lack of antigen and functional specificity, and shortage of numbers) can be overcome by extrinsically modifying cell-intrinsic properties. Heczey et al. recently demonstrated that introducing a CAR targeting GD2, a tumor-associated surface ganglioside, to sorted and expanded human iNKT cells can redirect their specificity independent of CD1d. Importantly, the CAR contained the 4-1BB signaling domain and biased the iNKT cells to a Th1 phenotype upon antigen engagement (98). This approach is an example of how to overcome the inherent limitations of iNKT cells. Conversely, CD1 and MR1 can be targeted by redirecting conventional MHC-restricted T cells with TCRs recognizing CD1/MR1-antigen complexes of interest. This would be one of the most practical and translatable methods of targeting CD1 and MR1 currently, since transducing T cells with recombinant TCRs is a fairly well-established methodology to redirect T cell reactivity and has been used in many trials (99). The major barrier to this would be to identify the appropriate TCRs capable of selectively recognizing diseased tissues, as discussed above, which will represent the rate-limiting step to target CD1 and MR1 through conventional T cells. Combining cell-based therapy targeting CD1 and MR1 with small-molecules (100), checkpoint blockade reagents, or other biologics could also prove to be beneficial. Anti-CTLA4 mAb treatment combined with adoptive HLA-restricted T cell therapy indeed demonstrated greater efficacy than adoptive T cell therapy alone (2). Lastly, CD1, MR1, or MHC-presented antigens, if they are expressed simultaneously or in combinations on the target cells, can be targeted together to minimize the immune escape routes of the malignant or infectious agents, since these three classes of antigen-presenting molecules likely present ligands derived from non-overlapping molecular pathways.

## Concluding Remarks

Adoptive cell therapy of MHC-restricted T cells has undoubtedly produced impressive clinical responses in chronically infected and cancer patients. Use of T cells targeting lipids and small molecule metabolites presented by CD1 and MR1 as a T cell graft will broaden applicability of T cell therapy to more diseases and patients without the limitation of HLA restriction. The research areas pivotal for successful adoptive CD1- and MR1-restricted T cell therapy, which are already underway, are to better characterize the pattern of expression of CD1 and MR1 molecules, identify disease-associated antigens processed and presented by CD1 and MR1 molecules, and isolate cognate TCRs or T cells with the desired function that recognize these antigen complexes but not others. Although the biology of CD1- and MR1-restricted T cells and their receptors require further study before being tested in clinical trials, they represent an exciting venue of therapeutic potential in the near future.

## Conflict of Interest Statement

The authors declare that the research was conducted in the absence of any commercial or financial relationships that could be construed as a potential conflict of interest.
